# Azimuthal multiplexing 3D diffractive optics

**DOI:** 10.1038/s41598-020-63075-8

**Published:** 2020-04-15

**Authors:** Haiyan Wang, Rafael Piestun

**Affiliations:** Department of Electrical, Computer, and Energy Engineering, University of Colorado Boulder, Boulder, Colorado 80309 USA

**Keywords:** Optical materials and structures, Micro-optics, Micro-optics, Optical manipulation and tweezers, Optical manipulation and tweezers

## Abstract

Diffractive optics have increasingly caught the attention of the scientific community. Classical diffractive optics are 2D diffractive optical elements (DOEs) and computer-generated holograms (CGHs), which modulate optical waves on a solitary transverse plane. However, potential capabilities are missed by the inherent two-dimensional nature of these devices. Previous work has demonstrated that extending the modulation from planar (2D) to volumetric (3D) enables new functionalities, such as generating space-variant functions, multiplexing in the spatial or spectral domain, or enhancing information capacity. Unfortunately, despite significant progress fueled by recent interest in metasurface diffraction, 3D diffractive optics still remains relatively unexplored. Here, we introduce the concept of azimuthal multiplexing. We propose, design, and demonstrate 3D diffractive optics showing this multiplexing effect. According to this new phenomenon, multiple pages of information are encoded and can be read out across independent channels by rotating one or more diffractive layers with respect to the others. We implement the concept with multilayer diffractive optical elements. An iterative projection optimization algorithm helps solve the inverse design problem. The experimental realization using photolithographically fabricated multilevel phase layers demonstrates the predicted performance. We discuss the limitations and potential of azimuthal multiplexing 3D diffractive optics.

## Introduction

With feature sizes comparable to electromagnetic wavelength, diffractive optics offers a unique pathway to light manipulation^1–3^. It expands the capabilities of conventional optics based on refraction or reflection and in conjunction with free-form^4,5^, graded index^6^, and artificial (meta) materials^7,8^ provide full access to the spatial degrees of freedom of light. Further, new insight in wave manipulation and the ever-increasing power of computers enable diffractive optics to generate user-defined wavefronts from arbitrary inputs, by virtue of degrees of freedom from pixels that can be addressed individually and independently^9–11^. Beyond classical applications such as beam shaping^12,13^, 3D display^14,15^, information security^16,17^, spectroscopy^18^, metrology^19^, and astronomical imaging^20^, emerging areas include optical tweezers^21,22^, novel microscopies^23,24^, coherent control^25,26^, quantum information^27,28^, neural networks^29,30^, and optogenetics^31,32^.

Three dimensional (3D) diffractive optics expand the capabilities of traditional two-dimensional elements not only by providing higher diffraction efficiency and higher information capacity, but also enabling functionalities such as multiplexing and space-variant functions^33–35^. The capability of controlling multidimensional spatial, spectral, temporal, and coherence functions of light fields is originated from the multi-subject nature of 3D diffractive optics involving diffraction, refraction, absorption, resonances, and scattering.

In spite of being a topic of great interest, 3D diffractive optics have not been fully investigated due to their physical and mathematical complexity, where the challenge stems from three aspects: First, the wavefront propagation must obey Maxwell’s equations, while most arbitrary target patterns do not, causing the problem to be inconsistent. Second, the finite degrees of freedom due to finite volumetric space-bandwidth and limited phase/amplitude modulation narrows the scope of possible solutions. Third, the interaction of novel physical phenomena within the volumetric optics and the need for multiplexing increasing information calls for mathematical models where multiple interdependent design metrics are optimized simultaneously. Moreover, it is often important to distribute the information evenly, within a relatively small volume under control.

Holographic multiplexing refers to the possibility of encoding multiple pages of data by changing spatial, frequency, or polarization characteristics of the inputs. It is a unique property of 3D diffractive optics which allows for independent information to be distributed throughout the recording medium. Individual signals can be retrieved with minimum crosstalk, as a result of the engineering of the volumetric refractive index structure^36^. The reconstruction degrades as the input beam deviates from the designed values, namely Bragg-like behavior, and this selectivity is mainly determined by the thickness of the structure^35^. Angular and frequency (wavelength) multiplexing are the most common forms of multiplexing. The former one enables additional information to be encoded but requires extra effort in alignment, whereas the latter one is easier to arrange but requires a complicated laser system that can be tuned in a broad spectrum^36^. Other techniques, proposed in optically recorded holography, include peristrophic^37^ and shift multiplexing^38^, referring to rotation and translation of the holographic sample. However, they are limited by the possible 3D fields obtained from the interference of an object and reference waves inside photosensitive materials.

Early in the 1970s, Alvarez and Lohmann independently proposed composite lenses whose focal length can be adjusted continuously by shifting laterally two optical elements with cubic phase profile^39,40^. Recent work improved on this idea by implementing the tuning mechanism through rotation^41,42^. These devices are designed analytically to continuously change the optical power of lenses or axicons.

In this paper, we propose azimuthal multiplexing, by which multiple output signals are switched upon the relative rotation of one or more layers within the 3D diffractive structure. We implement the inverse design with an iterative projection algorithm with distribution on layers^43,44^. We demonstrate experimentally the principle with two phase layers fabricated lithographically following the multilevel binary optics technique^45^. The paper is organized as follows. We first introduce the physical model to compute the light propagation in 3D diffractive optics by decomposing the device into multiple thin layers of phase modulation. We then present design results and experimental validation. We analyze the system performance and discuss the limitations.

## Theory

### Physical model

**Figure 1:**
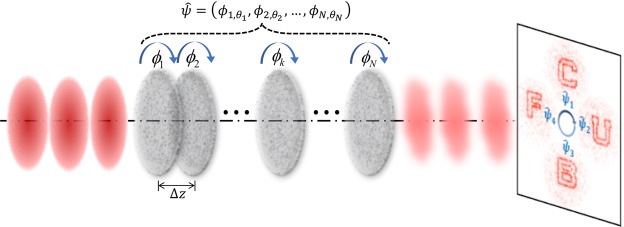
Physical model of 3D diffractive optics, which consists of N layers of phase modulation, separated by a short free-propagation distance $\Delta$z. Each layer can rotate with respect to the optical axis while optical waves are modulated upon propagation through the 3D diffractive optics. The state of the system is defined by the vector $\hat{\psi }$ composed of the orientation of each layer. The azimuthal multiplexing system is designed in such a way that different states generate different output functions.

We consider a stratified 3D diffractive optical element composed of multiple 2D layers of phase modulation spatially separated by thin homogeneous isotropic media (Fig. 1). Each layer can rotate with respect to a common axis (the optical axis). Under the scalar and thin-element approximation, the phase modulation for coherent illumination can be described by

1 $$E(x,y,{z}_{k}^{+})=\exp \{j{\phi }_{k,\theta }(x,y)\}E(x,y,{z}_{k}^{-}),$$

where *E* is the complex amplitude, *k* is the layer number and *θ* denotes its rotation angle. In the homogenous region between adjacent layers, diffraction occurs and can be described by the angular spectrum propagation in free space

2 $$E(x,y,{z}_{k+1}^{-})={{\mathscr{F}}}^{-1}\{{e}^{-j\sqrt{{k}_{0}^{2}-{k}_{x}^{2}-{k}_{y}^{2}}\cdot \varDelta z}\cdot {\mathscr{F}}[E(x,y,{z}_{k}^{+})]\},$$

where Δ*z* is the layer separation, “*z*^+^”, “*z*^*-*^” represent the coordinates immediately before and after the corresponding layer, and ${\mathscr{F}}$ is the Fourier transform. The wave field on the reconstruction plane is calculated using Fresnel or Fraunhofer propagation^46^.

3 $${E}_{R}(x,y)=\{\begin{array}{c}{{\mathscr{F}}}^{-1}\{{e}^{-j\sqrt{{k}_{0}^{2}-{k}_{x}^{2}-{k}_{y}^{2}}\cdot {z}_{R}}\cdot {\mathscr{F}}[E(x,y,{z}_{N}^{+})]\},\,{\rm{Fresnel}}\,{\rm{region}}\\ Q\left[\frac{1}{\lambda f}\right]V\left[\frac{1}{\lambda f}\right]{\mathscr{F}}[E(x,y,{z}_{N}^{+})],\,{\rm{Fraunhofer}}\,{\rm{region}}\end{array},$$

where *Q* is the quadratic factor and *V* is the scaling factor. *z*_*R*_ is the distance from the last layer to the reconstruction plane and *f* is the focal length of the Fourier lens. The propagation process is also reversible, described by the conjugate forms of the above equations. Accordingly, the phase for each layer satisfies

4 $${\phi }_{k,\theta }(x,y)={\rm{\arg }}\left\{\frac{E(x,y,{z}_{k}^{+})}{E(x,y,{z}_{k}^{-})}\right\}.$$

A 3D diffractive optics system is composed of *N* wavefront modulation layers that can rotate around a common axis. We define the vector $\hat{\psi }=({\phi }_{1,\theta 1},\ldots {\phi }_{N,\theta N})$ where ${\phi }_{i,\theta i}$ represents the orientation $\theta i$ of layer *i*. The system provides a different output functionality for different input states based on the information stored in the various modulation layers.

### Azimuthal multiplexing design

Suppose a 3D diffractive optics consists of *N* layers. In order to calculate the phase modulation function layer by layer, we start by setting all of them to random values while the amplitude is a circular function. The input is $E(x,y,{z}_{1}^{-})$ which contains the information of wavelength and incident angle, both are constants in this case. There are *m* + 1 pages of reconstruction patterns to be multiplexed ${E}_{R}^{0}(x,y)$, ${E}_{R}^{1}(x,y)$, …, ${E}_{R}^{m}(x,y)$, with the corresponding rotation angle of the *k*-th layer at 0, ${\theta }_{1}$, …, ${\theta }_{m}$. We design azimuthal multiplexing 3D diffractive optics using an iterative projection optimization algorithm with a distribution on layers method. This approach is flexible, as the layer being rotated can be any one or any combinations from 0 to *N*, and can switch during the encoding process.

We design the layer of phase modulation in round shape to ensure it rotates to an arbitrary angle without redundant pixels or the need to enlarge the beam size, and we apply bilinear interpolation to keep the number of active pixels constant during rotation. It is worth noting that the calculation of backward propagation should apply the following constraints to avoid error caused by zero denominator due to the zeros outside the aperture

5 $$\begin{array}{c}E(x,y,{z}_{k}^{-})=\sum _{i,j}E({x}_{i},{y}_{j},{z}_{k}^{-})=\frac{E({x}_{i},{y}_{j},{z}_{k}^{+})}{\exp \{j{\phi }_{k,\theta }({x}_{i},{y}_{j})\}}\\ {\rm{where}}\,{x}_{i},{y}_{j}\in circ\left(\frac{\sqrt{{x}^{2}+{y}^{2}}}{r}\right),\end{array}$$

where *r* is the radius of the layer. Hence, we establish the connection between the input $E(x,y,{z}_{1}^{-})$ and output ${E}_{R}(x,y)$ of the 3D diffractive optics.

Basically, there are three optimization loops embedded in the design algorithm. The first loop is to optimize one single layer according to the target metrics. We apply the forward propagating model described by Eqs. (1)–(3) from input to the wave field in front of the *k*-th layer $E(x,y,{z}_{k}^{-})$. We then continue to the reconstruction plane where the amplitude is updated with the pre-defined reconstruction field ${E}_{R}^{0}(x,y)$ and the phase is kept unchanged. Next, the backward propagation described by the conjugate form of Eqs. (1)–(3) is applied from the reconstruction field to the wave field after the *k*-th layer $E(x,y,{z}_{k}^{+})$. Thus the phase modulation can simply be derived from Eq. (4). We update ${\phi }_{k,0}^{0}$ and iterate until it reaches a satisfactory solution or a predefined number of iterations is completed. This is the first loop, which is repeated for all the remaining layers ${\phi }_{1,0}^{0}$, ${\phi }_{2,0}^{0}$,…, ${\phi }_{N,0}^{0}$.

We then rotate the *k*-th layer to ${\theta }_{1}$, and repeat the above process except the reconstruction field is ${E}_{R}^{1}(x,y)$. As a result, we obtain the phase modulation optimized for the second target ${\phi }_{1,0}^{{\theta }_{1}}$, ${\phi }_{2,0}^{{\theta }_{1}}$,…, ${\phi }_{k,{\theta }_{1}}^{{\theta }_{1}}$,…, ${\phi }_{N,0}^{{\theta }_{1}}$. We follow the same procedure until all the targets are encrypted in all the layers, and that becomes the second loop.

**Figure 2:**
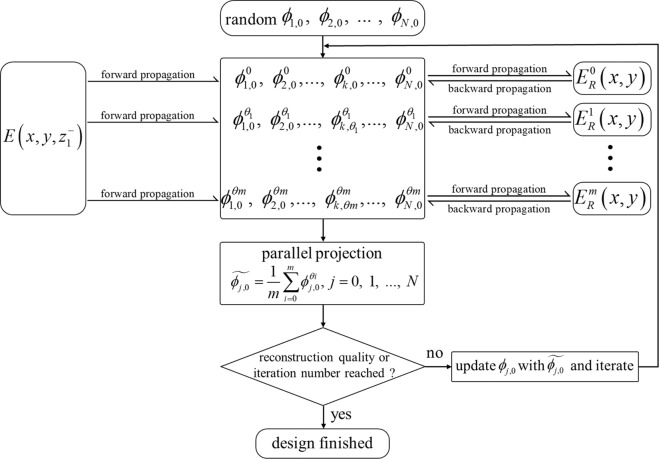
Flowchart of the iterative projection optimization algorithm used for the azimuthal multiplexing design. Within each iteration, the wave fields are forward propagated from the input to the plane right before the *k*-th layer to be designed, and backward propagated from the preset target to the plane right after the *k*-th layer to be designed.

To ensure all the multiplexing cases being considered are evenly distributed among all the layers, we apply a parallel projection, described by the following equation

6 $$\mathop{{\phi }_{j,0}}\limits^{ \sim }=\frac{1}{m}\mathop{\sum }\limits_{i=0}^{m}{\phi }_{j,0}^{\theta i},\,j=0,\,1,\,\mathrm{..}.,\,N,$$

and update the phase modulation functions. All the calculations up to this point conclude one iteration in the third optimization loop. The algorithm keeps iterating until the results are satisfactory or the iteration number is reached. The overall flowchart of the design algorithm is shown in Fig. 2.

It should be noted that the convergence of the algorithm depends on the task complexity, namely the number of functions to be multiplexed, and degrees of freedom available, namely the number of layers and number of pixels in each layer.

## Results

**Figure 3:**
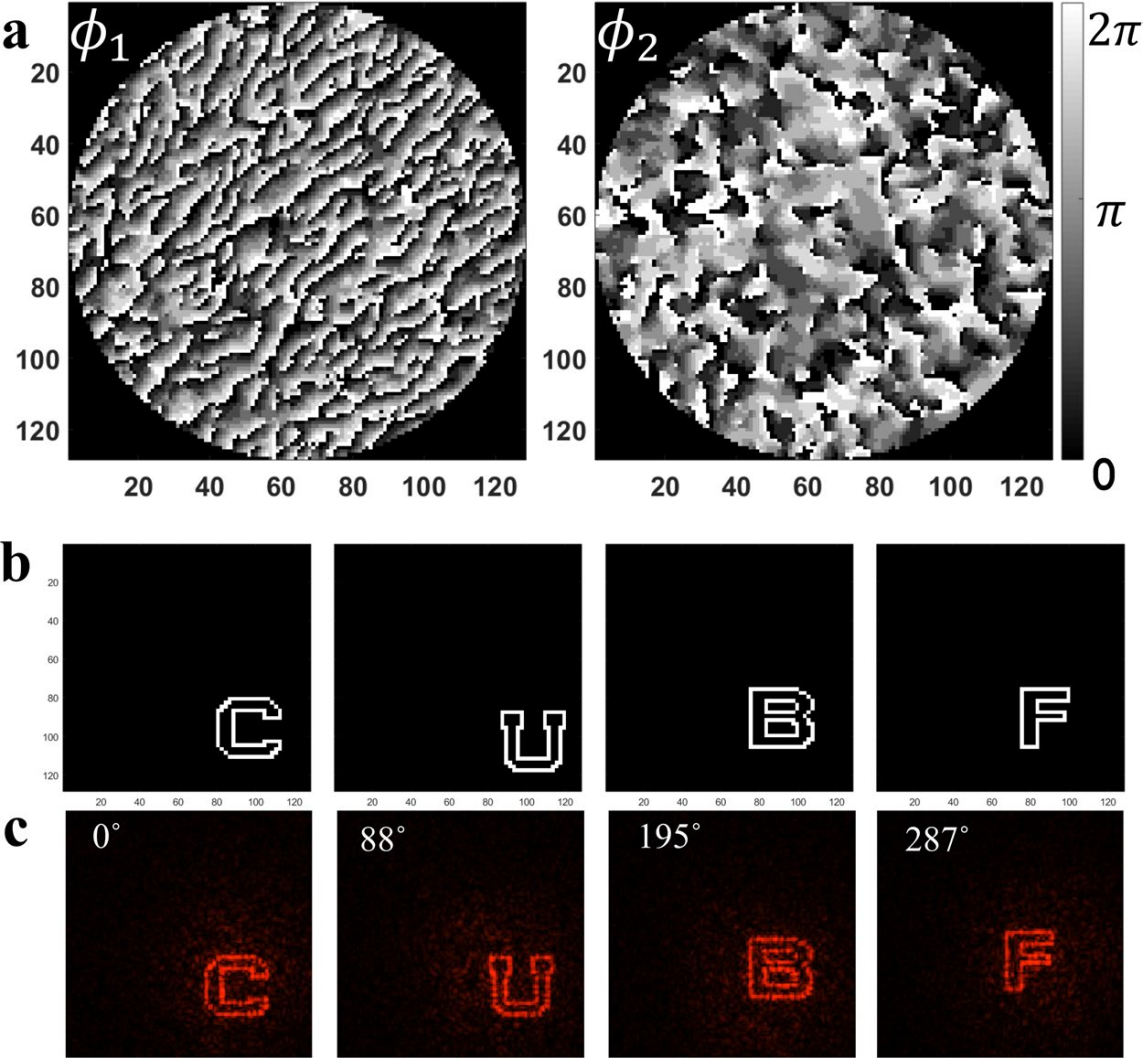
Simulation results of azimuthal multiplexing 3D diffractive optics. (**a**) The design of two layers of phase modulation discretized to 8 levels. (**b**) Target images of the 4 letters to be multiplexed. (**c**) Numerical reconstructions form the 3D diffractive optics while the second layer is rotated at angles specified by design.

To demonstrate the principle, we design a two-layer 3D diffractive optics for azimuthal multiplexing of 4 functions. The target patterns are arbitrary, and defined digitally on a computer as the letters “C”, “U”, “B”, “F” (Fig. 3b). They are encoded with 4 rotation angles of the second layer respectively, which are purposely chosen to be off multiples of 90° at 0°, 88°, 195°, and 287°.

### Design

The initial design of a 3D diffractive optics consists of two layers, separated by 1 mm. Each layer has 128 × 128 pixels with the pixel pitch of 40 µm. The parameters are chosen to facilitate the alignment in the experiment in the lab environment. If that is not a concern, with high precision mounts, more compact devices can be designed using smaller pixel size, and/or large volumetric bandwidth with enhanced pixel number.

Under a plane wave normal illumination at 633 nm, the two layers with continuous phase modulation ranging in 0~2π are designed using the algorithm described in the above section. However, to implement experimentally with photolithography, we need to discretize the phase distribution. Here we design it to 8 phase levels, because it simplified the experimental implementation without compromising too much efficiency. There are two approaches. The first one is the commonly used “hard-cut” approach where at each iteration the phase values are forced to the allowed ones they are closed to. The second one is the “soft-cut” approach which we built in our design algorithm. In this approach, we set regions centered in the allowed 8 phase values, and if the designed phase value is outside those regions, it will be expelled to the edge of whichever region it is closer to. The allowed regions shrink with the iteration number until, at the end, there are only 8 phase values allowed. Compared with the “hard-cut” approach, the “soft-cut” approach helps the algorithm’s convergence and improves the efficiency of the reconstruction. See Supplement 1 for detailed comparison.

Figure 3a shows the two designed layers with 8 phase levels using the “soft-cut” approach. The numerical reconstruction of the design with the second layer rotated at 4 encoded angles are shown in Fig. 3c (Visualization 1). Note that the reconstruction plane is enlarged twice (via zero-padding of the near field) to avoid wrap-up aliasing and to take light scattering into account. The diffraction efficiency of the 4 reconstruction patterns are 43.60%, 42.38%, 47.12%, and 44.83%, respectively.

### Experiment

In this section, we present experimental demonstration of azimuthal multiplexing with two-layer 8 phase level diffractive optics. To fabricate the DOEs, we first convert each of the two layers to three binary amplitude masks. Along with a circular aperture on a single wafer, they are fabricated using Heidelberg DWL 66 fs mask writer. The DOE substrate is an uncoated UV fused silica precision window (Thorlabs WG41010), with 1 inch diameter and 1 mm thickness. We first coat the substrates with 80 nm chromium using CVC thermal evaporator. Then we use chemical etching to take away the parts that define the aperture as well as the orientation. Next, we use the binary amplitude masks to fabricate the 8-level phase masks. The substrates are coated with AZ 4210 for 3 µm, a positive photoresist, on a spinner at the speed of 3000 rpm for 60 s. The photoresist is then pre-baked on a hotplate at 100 C for 90 s. During the exposure process on the SUSS MJB3 mask aligner, the pattern on the binary mask allow the photoresist on substrate to be exposed by UV light. That induces chemical change in the exposed region which is removed after developing with 1:3 concentration of AZ400K and de-ionized water. The last step is reactive ion etching (RIE), which is suitable for removing material along the vertical direction. Here we use a mixture of CF4 for 16 standard cubic centimeters per minute (SCCM) and oxygen for 4 SCCM, which yields an etching rate of 31 nm per minute. By proper control of the etching time, we obtain different etching depths for different exposures. The whole process is repeated 3 times for each phase mask and an 8-level modulation is achieved as a result. See Supplementary 1 for detailed fabrication process.

**Figure 4:**
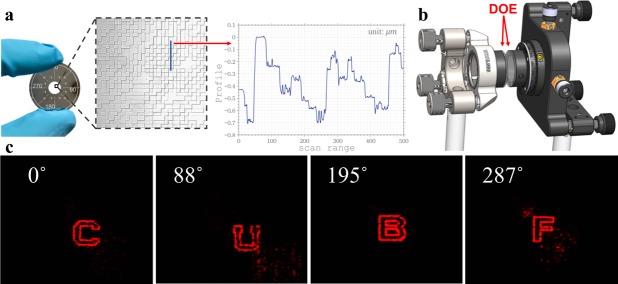
Experimental results of azimuthal multiplexing 3D diffractive optics. (**a**) One DOE fabricated using photolithography. The substrate is coated with chromium that contains markers on the periphery indicating orientation. The aperture encircles the layer of phase modulation at the center. The phase modulation is achieved by a 3-step exposure and etching. The plot shows a characterization of the surface profile using a 1D stylus profilometer. The diffractive optics is robust as the reconstructions can still be obtained with some roughness on the surface. (**b**) The setup for reconstruction. The two layers are attached on adaptors which are secured on lens mounts, with the etched sides facing each other. Both layers are adjusted normal to the incident beam, and separated by 1 mm. The layers are adjusted to be concentric by transverse shifts and can rotate with respect to each other. (**c**) Reconstructed images with threshold value 10% relative to maximum when the second layer is rotated, with respect to the first layer, at 0 °, 88°, 195°, and 287° (the angles specified by design).

Figure 4a shows the fabricated sample with a microscopic image of the surface after etching 3 times. We can see each etching step overlaps appropriately with the help of the alignment markers (See Supplement 1). The surface profile is examined with a 1D stylus profilometer, where 8 phase steps are recognizable. In spite of some roughness on the surface, which is probably due to the non-ideal condition of the RIE, the 3D diffractive optics samples are robust and the desired reconstructions are still successfully obtained as shown below. Figure 4b shows the reconstruction setup. We use industrial grade double-sided tape to attach the two substrates on adaptors, with the etched sides facing each other (the phase pattern of one layer is mirrored left-right in the fabrication process). The two adaptors are then secured on two lens mounts, one of which is mounted on a 3-aixs translation stage to provide control of tip/tilt and translation in X, Y, Z direction, the other provides control for the same as previous in addition to rotation (Thorlabs K6XS). Both layers are adjusted concentric and normal to the incident beam, with a separation distance of 1 mm as in the design. After the second layer, we place a Fourier lens with a focal length of 300 mm to yield a far-field plane of the output from the 3D diffractive optics, where a color CMOS sensor (Canon 5D Mark ii) is placed to capture the reconstructed images. With spatial-filtered and collimated illumination from a He-Ne laser, we obtained the reconstructed images (Fig. 4c) as the second layer is rotated to the designed angles. The measured diffraction efficiency is 33.65%, 29.28%, 36.46%, and 31.50%, respectively.

## Discussion

We use the design approach described in Sec. 2 to obtain 3D diffractive optics showing azimuthal multiplexing of 4 functions with 16 layers and each layer has 1024×1024 pixels (see Supplementary 1). Here, we investigate the diffraction efficiency as a function of the number of pixels and the number of layers, azimuthal selectivity and the smallest angular interval for multiplexing to avoid crosstalk.

**Figure 5:**
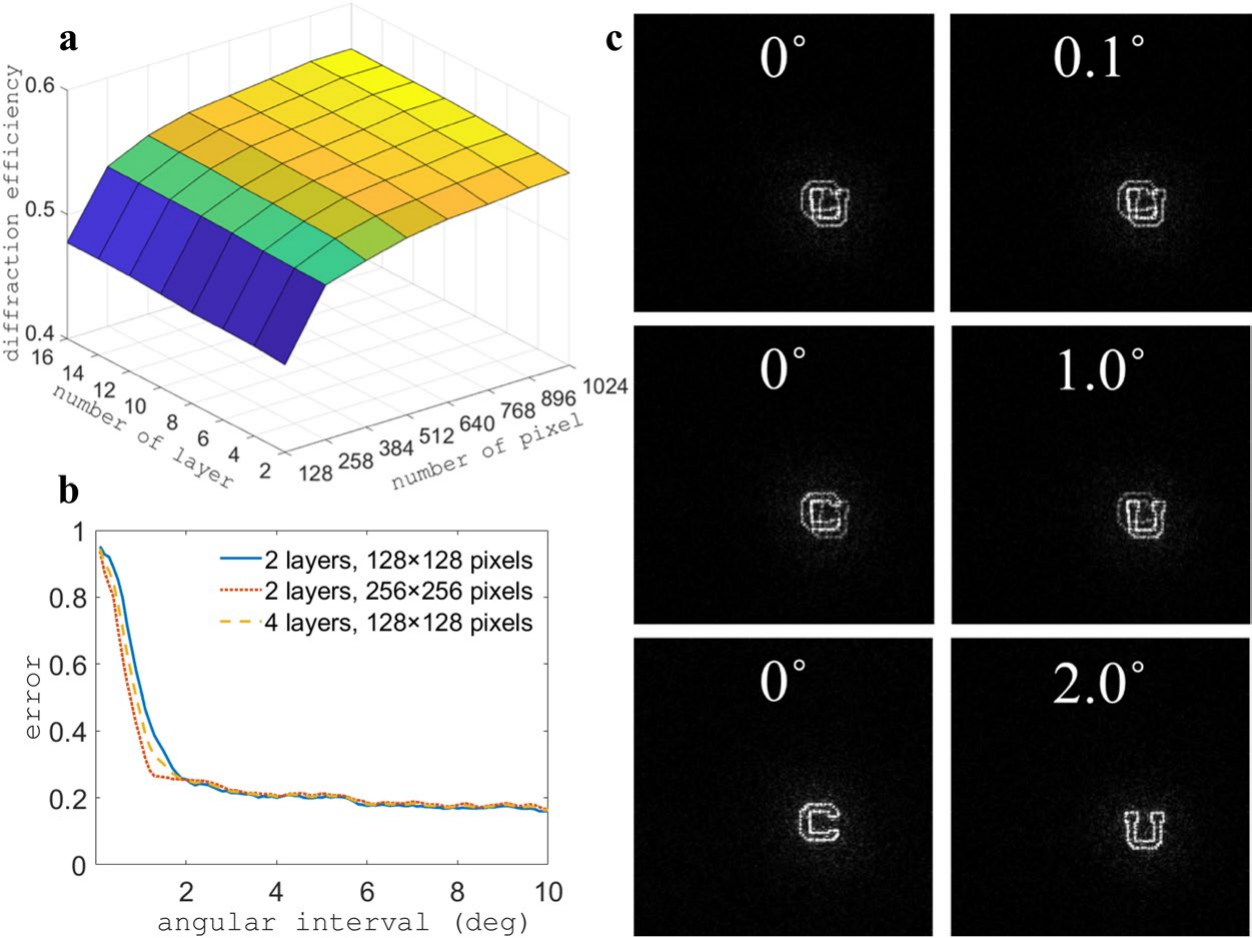
Scaling of azimuthal multiplexing 3D diffractive optics. (**a**) The mean of the diffraction efficiencies of “C”, “U”, “B”, “F” as functions of the number of layers and the number of pixels. (**b**) The relative error of “C” and “U” as a function of angular interval between the functions. Each point on the plots corresponds to a different design. (**c**) Simulated reconstructions for angular intervals equal to 0.1°, 1°, and 2°, showing progressive reduction of crosstalk.

First, we study numerically the effect of system parameters on the diffraction efficiency (DE). The diffraction efficiency is defined as the ratio of the intensity in the target area to the intensity of the input beam. We multiplex 4 functions representing the letters “C”, “U”, “B”, “F”. We change the number of layers from 2 to 16 and the number of pixels in each layer from 128 to 1024. We record the mean of the 4 diffraction efficiencies and the result is shown in Fig. 5a. The diffraction efficiency is enhanced by either increasing the number of layers or the number of pixels. However, the rate of improvement of the DE becomes slower with larger parameters, and saturates at some point. A longer computational time is also required when increasing the number of layers or the number of pixels.

Second, we investigate the minimum angular interval to avoid crosstalk. We apply the azimuthal multiplexing scheme to two functions, namely the letters “C” and “U”. We change their angular interval from 10° to 0.1°, at decrements of 0.1°. We use the relative error, defined as the ratio of the intensity outside the target area to the one in the target area, to evaluate the quality of the reconstructions. The result is shown in Fig. 5b. The error is higher as we decrease the angular interval, with no crosstalk, partial crosstalk, and complete crosstalk showing in Fig. 5c. We obtain a smaller angular interval without crosstalk by either increasing the number of layers or the number of pixels.

**Figure 6:**
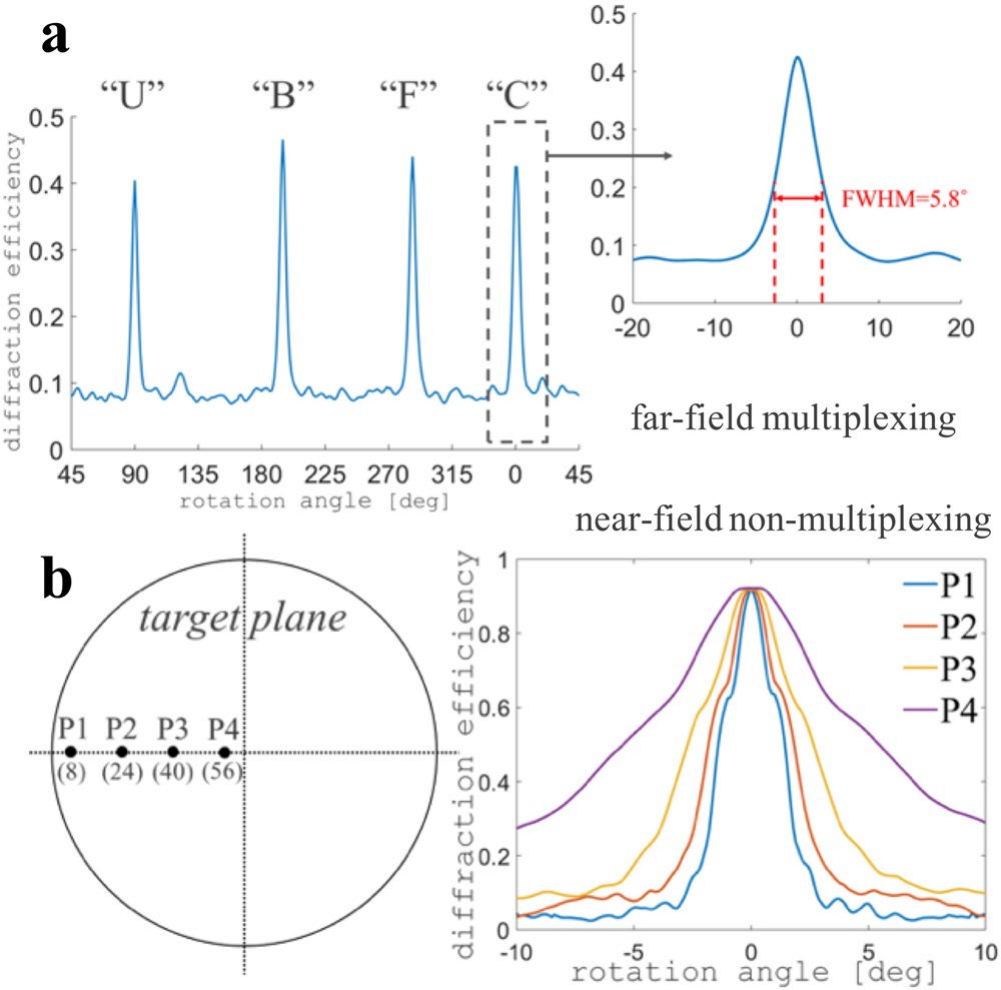
Azimuthal selectivity of 3D diffractive optics. (**a**) The diffraction efficiency as a function of rotating angle of the second layer with respect to the first layer. The FWHM of each single reconstruction patterns are between 5° to 6°. (**b**) The azimuthal selectivity in the near-field for 4 point targets located radially on the target plane. The point closer to the edge has better selectivity than the one close to the cent, indicating the azimuthal selectivity in the near-field depends on target location.

Azimuthal multiplexing is an important functionality enabled by the proposed 3D diffractive optics. The azimuthal selectivity is the angular interval where the reconstructed patterns are still recognizable. A direct sense of multiplexing system performance can be perceived in Visualization 1. For the design described above, we rotate the second layer 360° with respect to the first layer, and record the diffraction efficiency of the 4 encoded patterns around their design angles, as shown in Fig. 6a. The FWHM of one reconstruction is between 5° to 6°.

We also investigate the selectivity in the near-field, in the non-multiplexing case. To simplify, we use 4 points as the targets. They are located at the 8^th^, 24^th^, 40^th^, and 56^th^ column in the 128 columns target plane. The parameters of the 3D diffractive optics are the same as previous case, except the reconstruction is at 50 µm after the second layer. The selectivity of the 4 points as the second layer rotates in a ±10° at the increment of 0.1° is shown in Fig. 6b. We find the azimuthal selectivity in the near-field depends on the location of the target, i.e. the targets closer to the edge have better selectivity than the ones closer to the center. The reason is that as the layer rotates, the induced pixel changes are proportional to the radial values.

## Conclusion

In this report we introduced the concept of azimuthal multiplexing and demonstrated an approach to design and implement it with 3D diffractive optics. This is achieved by a stratified DOEs layout with iterative optimization algorithms. As a result, arbitrary optical information can be encoded azimuthally in the 3D diffractive optics and retrieved by rotating part of its components relative to the others. As a special case, one can encode different functions by rotating the input wavefront relative to a diffractive optics device. The designs are not based on weakly scattering or the Born approximation enabling for multiple forward scattering events while neglecting the weak backward scattering. This enables higher flexibility and efficiency through the use of high index contrast diffractive layers. The fundamental opportunities and limitations were analyzed, while the experiments using photolithography confirmed the predicted performance.

The results further show that extending diffractive optics from two dimensions to three dimensions enables new multiplexing opportunities. Rather than the traditional use of cascaded diffractive optical elements to encode amplitude and phase, our proposed layered 3D diffractive optics is a computationally designed volumetric structure that enables multiplexing. This is the result of multiple independent spatial mode channels being established between the input and the output of the system, reducing the dimensional mismatch essential to the control of light fields in multiple dimensions (spatial, spectral, temporal, or coherence function). The approach also provides a different perspective on 3D diffractive optics design and further contributes to the inverse problem community by solving the nonlinear inverse problem to achieve a given task using azimuthally rotating phase layers.

A number of applications of azimuthal multiplexing 3D diffractive optics can be envisaged that require switching of different outputs by rotation of one layer or the input field. For instance, information security is a critical issue in optical communication network systems to prevent data acquisition from unauthorized personnel. Hence, the proposed azimuthal multiplexing could be applied in information encryption and authentication. The complexity of deciphering the code would increase exponentially as more layers are employed in the 3D diffractive optics. In a different application, it is intriguing to analyze the relation between azimuthal multiplexing and the generation of beams with orbital angular momentum associated with azimuthal phase functions. Such beams have been applied in optical trapping^47^, quantum key distribution^48^, optical communications^49^, and stimulated emission depletion microscopy^50^.

## Supplementary information

Supplementary Information.

Visualization 1.

## Data Availability

The data that support the plots within this paper and other findings of this study are available from the corresponding author upon reasonable request.
