# Supplementary material for: Azimuthal multiplexing 3D diffractive optics
Source: Sci Rep. 2020 Apr 15;10:6438. doi: 10.1038/s41598-020-63075-8 (PMC7160109; doi:10.1038/s41598-020-63075-8)
Supplement: Supplementary file 1 — Supplementary Information. [file 41598_2020_63075_MOESM1_ESM.pdf]

Azimuthal multiplexing 3D diffractive optics: supplementary material

HAIYAN WANG[1] AND RAFAEL PIESTUN[1]

[1]Department of Electrical, Computer, and Energy Engineering, University of Colorado Boulder, Boulder, Colorado, 80309, USA
**hawa8774@colorado.edu*

Abstract: This document provides supplementary information to “Azimuthal multiplexing 3D diffractive optics,” https://doi.org. It presents additional information on expansion of the design method to large volumetric 3D diffractive optics, comparison of different phase discretization methods, and detailed description of the photolithography fabrication method used in the experiment.

Demonstration of design with large volumetric space-bandwidth

In this section, we show the proposed design method can be extended to obtain results for large volumetric space-bandwidth devices. We design 3D diffractive optics consisting of 16 layers, with 1024×1024 pixels in each layer. The device presents azimuthal multiplexing of four functions representing the letters “C”, “U”, “B”, “F”, each appearing when the last layer is oriented at 0˚, 88˚, 195˚, and 287˚. Fig. S1.a shows the 16 design phase patterns with 8 quantization levels. The calculation is completed within 24 hours on a PC with Intel-Core i7-8700 CPU at 3.2GHz and 16GB RAM. The diffraction efficiencies are improved to 55.95%, 56.32%, 60.46%, 52.50% for each reconstructed pattern (Fig. S1.b).

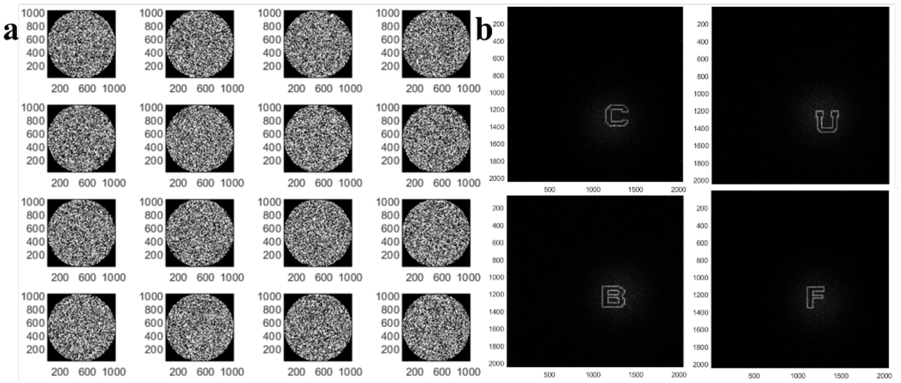

Fig. S1. Demonstration design of azimuthal multiplexing 3D diffractive optics with large volumetric space-bandwidth product. **a** Azimuthal multiplexing 3D diffractive optics with 16 layers and 1024×1024 pixels in each layer. **b** Reconstructed patterns when the last layer is rotated at 0˚, 88˚, 195˚, and 287˚.

Generation of gray level objects and phase objects

In this section, we show the proposed class of devices is capable of generating gray objects and phase objects. We encrypted two images, Mona Lisa and Da Vinci, each consisting of 256×256 pixels. The 3D diffractive optics has two layers with 512×512 pixels in each layer, and it is

designed in such a way that when the second layer is at its original position, the profile photo of Mona Lisa shows up, while when the second layer is rotated clockwise by 90˚, the profile photo of Da Vinci shows up. The reconstructed images are shown in Fig. S2a. The speckled images originated from the nature of computer-generated 3D diffractive optics of diffusively scattering objects[1].

In general, the proposed approach is capable of generating phase objects as well. However, it should be noted that for phase objects, the condition for the convergence of the algorithm is often stricter, namely harder to achieve due to the lower number of free parameters available once the amplitude and phase are imposed. The reason is that when imposing amplitude constraints, the phase is a free parameter, while also the amplitude outside the target area is a free parameter. Both conditions help the algorithm converge. When the phase is the constraint, the amplitude is uniform by default, which means there are fewer free parameters. Here, we encode a uniform amplitude in a small window around the letter "C" and "U", where the phase values on the two letters are 1.5π and 0.5π, respectively. That information is encoded in two layers of the 3D diffractive optics with 128×128 pixels in each layer, and can be switched depending on the orientation of the second layer (the phase letter "C" shows up when the second layer is at its original position, while the letter "U" shows up when the second layer is rotated clockwise by 90˚). Fig. S2b shows the phase value on the reconstruction plane for the two cases.

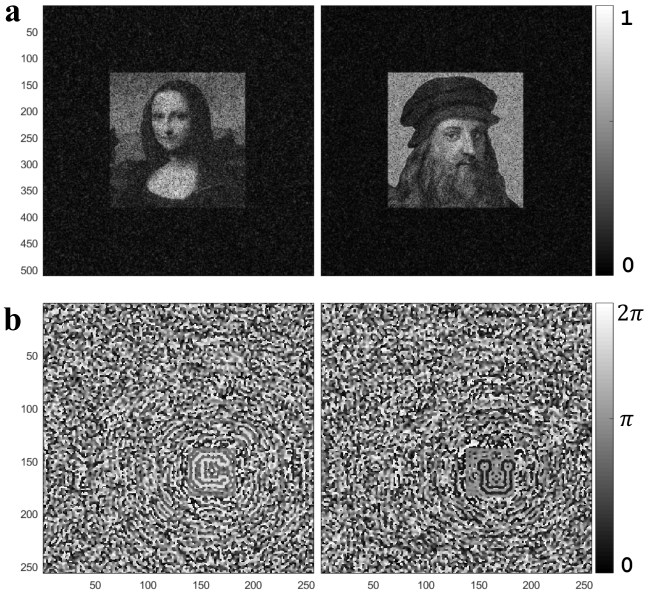

Fig. S2. Demonstration of generating gray object and phase object. **a**. Two gray images are multiplexed by the 3D diffractive optics with the proposed method. **b**. Two phase images are multiplexed by the 3D diffractive optics with the proposed method.

Diffractive optics implementation methods overview

There are various ways to implement the designed DOEs. A widely used method is via spatial light modulators (SLMs), which allows for easy and fast display of phase patterns with 8 bit resolution (256 grey levels) on the liquid crystal panels. However, SLMs are polarization sensitive thus suffer from substantial loss of energy[2]. Moreover, they are 2D devices thus not suitable for implementing 3D diffractive optics unless cascaded or creating a folded system[3]. Alternative methods generate surface relief structures in an optically transparent material such

as glass, quartz, or polymer, and include ion beam etching, direct diamond tuning, gray-level lithography, and multi-step binary exposure photolithography[4].

In our experiment, each layer of the 3D diffractive optics is fabricated using a photolithographic method. To approximate a continuous surface profile, the lithography process is repeated M times to yield a discrete structure with 2^M steps. Here we use 8 phase levels and accordingly 3 amplitude masks are needed for the fabrication. The phase modulation induced by the depth change is given by

$$h(x,y)=\frac{\lambda}{n(\lambda)-1}\frac{\phi(x,y)}{2\pi}, \tag{S1}$$

where λ is the wavelength of operation and n is the refractive index.

Phase quantization methods

To prepare for the fabrication, we need to discretize the phase values in the design. In particular, we allow 8 etching depths which correspond to phase values of 0, 1/4 π, 1/2 π, 3/4 π, π, 5/4 π, 3/2 π, and 7/4 π. One common quantization method is “hard-cut” where at each iteration the phase values are compelled to the closest allowed values. Here, in contrast, we use a “soft-cut” method to improve the coding. In this method, during the iterative optimization, the design phase values are allowed in a region around the preset values. These regions shrink after each iteration until only 8 phases are allowed in the end. In order to evaluate the performance of this phase discretization method, we design azimuthal multiplexing 3D diffractive optics consisting of two layers, with 128×128 pixels in each layer. In one case, we multiplex two functions, namely “C” and “U” with 0˚ and 90˚ of the rotation angle of the second layer. In another case, we multiplex four functions of “C”, “U”, “B”, “F” with 0˚, 88˚, 195˚, and 287˚. The diffraction efficiencies are shown in Fig. S3.

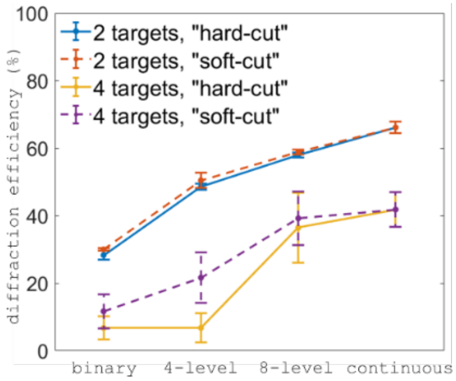

Fig. S3. Comparison of diffraction efficiency of azimuthal multiplexing 3D diffractive optics designs using different phase discretization methods and number of targets to be multiplexed.

Photolithography method

With the soft-cut method imbedded in the design algorithm, we obtain two layers of azimuthal multiplexing 3D diffractive optics. Each of the two layers has 8 phase levels, thus requiring 3 binary masks, as shown in Fig. S4a. The three binary masks are then arranged together with another mask defining the aperture and orientation angles on a single wafer with a size of 4 inch by 4 inch (Fig. S4b). It should be pointed out that the second layer is horizontally inverted, since we etch the two substrates on the edge and place them facing each other during the

reconstruction. It is also crucial that the three binary masks are aligned at the exact same location during the exposure process.

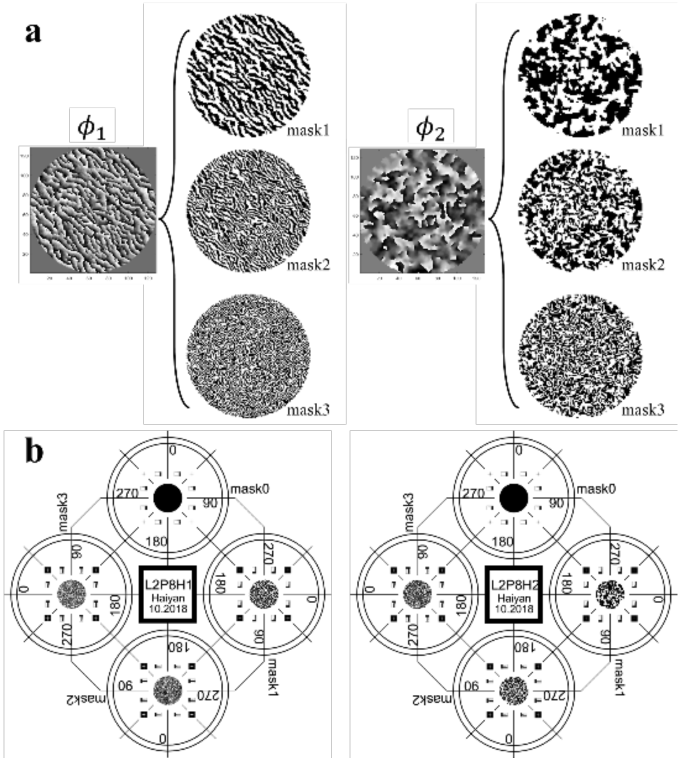

Fig. S4. Binary amplitude mask designs for 3D diffractive optics. **a** Each of the two 8-level phase masks are generated by three binary masks. **b** The three binary masks are arranged on a same wafer with another mask defining aperture and orientation angles. The design is fabricated using Heidelberg 66FS mask writer.

To facilitate that, several alignment markers, coarse and fine, are embedded around the modulation area. The wafer is fabricated with a Heidelberg DWL 66FS (Fig. S5a), and the examination under microscope shows good quality structures (Fig. S5b). The microscopic images of the sample after each etching shows precise alignment of binary masks during each exposure (Fig. S5.c).

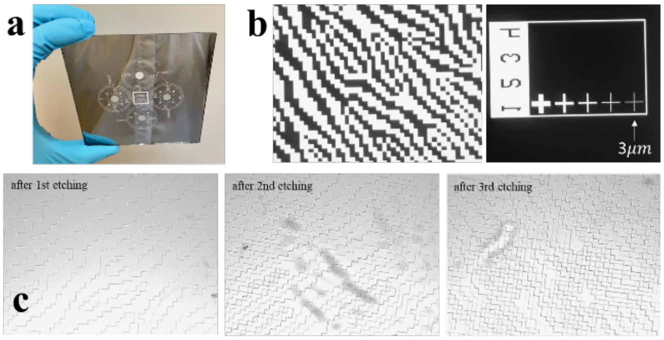

Fig. S5. Fabrication of diffractive optical element using photolithography. **a** Binary masks fabricated by Heidelberg 66FS mask writer. **b** Microscope images of the fabricated binary masks. The binary masks are aligned well during each exposure/etching. **c** Microscope images of the substrate after each of the three etching processes.

References

1. Wyrowski, F. & Bryngdahl, O. Speckle-free reconstruction in digital holography. *JOSA A* **6**, 1171–1174 (1989).
2. Pavani, S. R. P. & Piestun, R. High-efficiency rotating point spread functions. *Opt. Express* **16**, 3484–3489 (2008).
3. Wang, H. & Piestun, R. Dynamic 2D implementation of 3D diffractive optics. *Optica* **5**, 1220–1228 (2018).
4. O'Shea, D., Suleski, T., Kathman, A. & Prather, D. *Diffractive Optics: Design, Fabrication, and Test*. (SPIE PRESS BOOK, 2003).
